# An Exciting Stretch in the History of Investigación y Educación en Enfermería*

**DOI:** 10.17533/udea.iee.v41n3e15

**Published:** 2023-10-31

**Authors:** Clara Inés Giraldo-Molina

**Affiliations:** 1 Nurse, M.Sc. Full Professor, Retired. Facultad de Enfermería de la Universidad de Antioquia, Colombia. Director of Investigación y Educación en Enfermería from 2001 to 2004. Email: clara.giraldomolina@gmail.com Universidad de Antioquia Facultad de Enfermería Universidad de Antioquia Colombia clara.giraldomolina@gmail.com

Constructing knowledge in nursing and disseminating it has always been the commitment and obsession of the Faculty of Nursing at Universidad de Antioquia, with the pledge to contribute to enriching the discipline and profession. Obsession that achieves its goal only through the rigorous, devoted, and articulate work of many people, and in the case of the journal *Investigación y Educación en Enfermería* by the authors, referees, editorial boards; style reviewers, editing consultants, illustration guides, monitors and typists, to mention only some.

As indicated by the directors who preceded me in the post, *Investigación y Educación en Enfermería* flourished during the early 1980s as a scientific publication of the Faculty, notwithstanding the difficulties derived from the recent incursion by Nursing into the scientific world of our country. For everybody, it is known how in Colombia this esteemed profession was considered a task dependent on and subject to medicine, even during the second half of the 20th century, when research work by nurses was starting to become visible, difficulties added to stumbles of economic nature encountered when needing to edit and publish texts with academic and scientific quality.

But, due to the tenacity of deans, directors, and editors *Investigación y Educación en Enfermería* managed not only to rise, keep with its two annual editions, and remain at a high level over time, but also stand out in the universe of the country’s scientific publications. As a result of such difficult task, it was accepted in the ranking by COLCIENCIAS and in 1996, it reached category A of said demanding classification as a reward to the arduous work by the then director, my dear friend and colleague Luz Ángela Ramírez J.

Classification that, besides representing joy for the Faculty and the University, meant a challenge for those of us who assumed the Journal’s direction, like keeping it in the country’s index of scientific publications defined by COLCIENCIAS and known as PUBLINDEX, which for each For each call, the level of demand of the categories of the ranking increased.

In this order of ideas, it is worth noting how scientific journals, over time, went from being entities of particular dissemination of the scientific and academic production of professors from the Faculties of each publishing institution, to being publications where it was an imperative of scientific quality the international participation of authors, referees, members of advisory boards, and publishers, who, in addition, had to have an academic and scientific career demonstrated by their graduate training and by their recent articles in journals recognized in national and international contexts.

Another characteristic of PUBLINDEX consisted in that, for the most demanding categories, the number of scientific quality articles was periodically expanded, which always meant an important effort for journal directors or editors to collect, for each issue, a significant number of high-quality scientific articles, and, which - furthermore - obtained the endorsement from qualified and undoubtedly very demanding referees.

But the difficulty was established because the modification of criteria was announced at the time of the call, that is, a few days prior to submitting all the information of the publication to PUBLINDEX - and not at the beginning of the call period, two years before -making it impossible to comply with some criteria in the numbers already edited of the journals that would enter the call; inconveniences that led to disharmony among the PUBLINDEX directors and directors and editors of the University's journals and, even from other institutions in the country. This meant that it was possible that, despite the conditions of the publications in terms of the number of high scientifically demanding articles and other academic and administrative criteria, their position in the ranking would be maintained, or worse still, they would descend a level. That is, staying in a category meant having successfully improved the quality of the publication, a matter difficult to understand by those who were not direct participants in those processes.

Consequently, during this period, moments of unrest emerged among editors and directors of the University's journals, motivating the reconstitution of the Institution’s Committee of Editors of Scientific Publications, which for a considerable time - over three years - had stopped meeting. This collegiate body, during 2003 and 2004, undertook the intense task of preparing a proposal to update the regulations of the Alma Mater's serial scientific publications. A regulation represented in a Rectoral Resolution draft aimed at replacing Superior Agreement 108 of September 1988, and by which the policies and regulations for the Institution’s academic journals were proposed in such a way that an updated regulatory instrument was available for the administrative management of serial publications, and to achieve greater financial and editorial support from the University.

Also worth mentioning is that the call for indexing of scientific journals, which COLCIENCIAS published every two years, appeared on its website precisely when we were approaching the vacation period and, thus, began a tortuous journey of immense suffering aimed at collecting, at any cost, information required by the Index, data mostly found in the authors of the articles, referees, and other collaborators and that we only obtained after many attempts. Thereby, the individuals working in the IEE Journal at the time, Alex Gómez, enthusiastic and committed monitor; Ana Lucía Noreña, professor assistant to the director, who focused her interest and responsibility on the objectives of the publication; and the director were the last to leave for vacation, however, with the satisfaction of having sent whatever was necessary to participate in the call again.

Despite the vicissitudes, it was a pleasant job thanks to the efforts of the entire team during the period in question; *Investigación y Educación en Enfermería* remained in the COLCIENCIAS Index, given that it obtained the ranking in 2001 and 2003. The last ranking was recognized by members of the Institution’s central administration, when in 2003, as director, I was selected by the Vice-rector for Research to be a member of the "Fund Committee to support scientific journals and scientific dissemination" as advisor for the Committee for the Development of Research (CODI, for the term in Spanish) at Universidad de Antioquia. Likewise, the director for “IATREIA”, the journal of the University’s Faculty of Medicine requested support and accompaniment from the IEE director to position said journal in the COLCIENCIAS Index.

Now, the day-to-day life of a director included multiple activities, not necessarily simple, like receiving articles for possible publication, controlling the article’s compliance with criteria, reading it, identifying and selecting referees, collaborating peers from the country and from the international scientific community. Perhaps among the most delicate tasks was the need to contact possible referees - who in line with the requirements had to be “sage” and, therefore, very busy individuals - request their collaboration in revising the article and ask them to do so within a reasonable time, all without any other remuneration than their recognition as members of the publication’s group of referees; in addition to the precarious economic conditions available to manage the journal.

Likewise, permanently, as the publication's director, I had to invite personalities from the scientific world of Nursing and Health to participate, either as authors or evaluators, or as participants in the editorial and advisory boards. Also, I had to be aware of developments in the world's scientific publications, for which it was advisable to look for ways to be part of the groups and networks of editors in Nursing and Health, seeking to solidify and enhance the development of our publication.

In our journal’s internationalization process, we carried out actions on several fronts, like agreements with Universidad de Alicante, Spain, and with Universidad de Guadalajara, Mexico, conceived to co-edit texts of academic interest for undergraduate and graduate training, in addition to a contract to translate research books with Sage Publishing House in the United States, as explained ahead. Participation in the meeting of directors of Ibero-American nursing journals began with support by Professor José Ramón Martínez, from Universidad de Alicante, member of our group of collaborators. Another matter of international projection that brought us joy was the recognition of *Investigación y Educación en Enfermería* among the first 10 scientific nursing publications in Latin America, given the quality of its articles.

Recreating the Journal’s pages with artistic illustrations was another obstinacy conquered due to support from the University Museum embodied by the director during said period, Dr. Roberto León Ojalvo, and the curator of the visual arts section, Mauricio Hincapié, who carefully reviewed the content of each issue to guide me in the selection of possible images that could harmonize with the theme of each article and the most relevant illustration for the cover of the journal.

Particularly, being the director of *Investigación y Educación en Enfermería* meant, besides being responsible for the functions of professor and researcher in the Faculty, to assume the coordination of the Editorial Project that had begun during the direction of my colleague and friend Amparo Zapata Villa. A project created to address one of the weaknesses found in the self-evaluation process for accreditation purposes of the Faculty's Nursing Degree Program, conducted in 1998, which can be inferred from the self-evaluation report of that time that found a scarce average number of scientific publications by the faculty group, “observations not published”.

In turn, one of the purposes of the celebration of twenty years of *Investigación y Educación en Enfermería*, in 2003, consisted in the edition of a special offprint of the publication with the Cumulative Index of *Investigación y Educación en Enfermería*: 1983 - 2003, which presented the information in three sections, Author Index, Title Index, and Subject Index. This work was possible due to the commitment by the then director of the Faculty’s Library, Olga Inés Gómez, who together with librarian Marta Cecilia Galeno, organized and categorized the articles published from 1983 to 2003.

This beautiful task of directing a journal also brought moments of mistakes and difficulties, some salvageable through the support from the department's directives and from professors committed to nursing knowledge; others insurmountable, such as weaknesses in the motivation to subscribe to the journal by a large number of professors, or insufficient application of articles from the Journal in teaching and professional tasks, that is, due to its lack of use as an important source of learning and due to the scarce proposal of articles of their authorship for possible publication. Nevertheless, difficulties were offset by many moments of rejoicing generated by the Journal’s ongoing developments and achievements.

One of the most pleasant successes for the direction of the publication was the creation of a new section in the journal, called “Encounters and Disagreements (*Encuentros y Desencuentros*) in the experience of caring”, which arose as a response to the encouragement by Professor Carmen de la Cuesta and in harmony with a commitment by the Faculty, as it was, and continues to be, to strengthen “Care” as an academic object, but also from professional practice. In this sense, IEE was created to disseminate care experiences, whether they had been appropriate or, on the contrary, unwise, while in both cases it would be supported with theoretical and academic argumentation supported by professors in charge of the respective reflection according to the theme of the specialty. It was sought for professors to take advantage of this section to apply it in learning experiences about care and its significance in everyday life.

This new space had a tutor on her own initiative, Professor María Eugenia Molina, who, identified with the importance of this publication space, practically took on the task of encouraging students to dare to write care experiences that would have been significant for them. Professor Molina was in charge of the academic or ethical reflection of many of the experiences published in this section during the period of the story. Thus, *‘Encuentros y Desencuentros’* became a permanent space of the journal dedicated to our disciplinary and professional ideal.

The agreement with Sage Publishing in the United States consisted in translating from English to Spanish four qualitative research texts: “Basics of Qualitative Research, Techniques and Procedures for Developing Grounded Theory”, by Anselm Strauss and Juliet Corbin; “Critical Issues in Qualitative Research Methods”, by Janice M. Morse as editor; “Making Sense of Qualitative Data: Complementary Research Strategies” by Amanda Coffey and Paul Atkinson; and “Writing Up Qualitative Research”, by Harry Wolcott. 

Sage Publishing agreed that the translation would be conducted as long as there was an academic review of the translation by Professor Carmen de la Cuesta, an expert in qualitative research. This meant judiciously reading each of the translated texts, a task in which Professor Gloria María Franco and myself accompanied Professor Carmen, a pleasant and professionally enriching experience. The presentation or delivery of these four texts to the academic and research community had the participation of one of the authors, Harry Wolcott, who, besides accompanying us at the academic event, led a workshop on the topic of his book aimed at the University’s research professors.

Being director of our journal and of the editorial project represented a marvelous adventure, because in addition to what has been reported, the demand of being in front of a computer for hours on end, allowed me to travel through virtual space and venture into different contexts and countries that offered peer publications with immeasurable promise, full of opportunities that warranted consideration in our publication. Also, this experience provided the possibility of meeting and interacting with members of the global academic and scientific nursing and health community, a group engaged in building knowledge for health care, so desired and necessary for professional practice.

The University’s Department of Publications was a fundamental partner for the editorial project; several courses on writing to publish aimed at the Faculty’s professors were carried out with people from the department, which had to conclude with an essay or text proposal for possible publication once writing was completed. Texts of academic interest for the Faculty were also published, along with the editing of four texts on qualitative research translated within the framework of the agreement with Sage Publishing.

Recently, I was reviewing documents of interest to nursing and particularly on the occasion of the reports of the ICN Congress held in Montreal from June 30 to July 1 of 2023, in its publication “Charter for change: Our nurses, Our future”, once again the importance of nursing professionals is recognized in providing care and for the leadership they exercise in global health systems; essential personnel to achieve healthy communities, however, the invisibility of nurses is reiterated, despite progress and developments of the profession. In this regard, I believe nursing professionals continue with the challenge of making our work and professional achievements visible; among many actions, we must promote scientific publications, disseminate in an academic manner and with scientific support our teaching experiences, research and professional practice. 

I do not want to end without recognizing the permanent support for the edition of the Journal by institutions and companies, which, through their advertising and during the entire period of my administration, contributed significantly to the financial part. In this sense, Tena, Anec Seccional Antioquia, Ecosesa, and Corpaúl, among others, were unwavering partners of this academic and editorial purpose.

My everlasting gratitude to all those allies and accomplices of this extraordinary academic and professional experience.

Note: the foregoing narrative is supported in the editions of the journal issues from September 2001 to March 2004.

